# Assessment of the Efficacy and Safety of a New Treatment for Head Lice

**DOI:** 10.5402/2012/460467

**Published:** 2012-10-30

**Authors:** Sophie Mac-Mary, Rafat Messikh, Adeline Jeudy, Thomas Lihoreau, Jean-Marie Sainthillier, Bernard Gabard, Catherine Schneider, Philippe Auderset, Philippe Humbert

**Affiliations:** ^1^Skinexigence, Pavillon Sainte-Lucienne, Saint-Jacques University Hospital, 25030 Besançon, France; ^2^Research and Studies Centre on the Integument (CERT), Department of Dermatology, Saint-Jacques University Hospital, University of Franche-Comté and INSERM UMR1098, SFR FED 4234 IBCT, 25000 Besançon, France; ^3^Dermlink International Ltd., Grosvenor Gardens House, 35/37 Grosvenor Gardens, London SW1W 0BS, UK; ^4^Rausch AG, Bärenstraße 12, 8280 Kreuzlingen, Switzerland

## Abstract

Infestation with head lice is a widespread, persistent, and recurring issue leading to serious health problems if untreated. We are facing resistance phenomena to usual pediculicides and questions about their direct or cumulative toxicity. The aim of this trial was to assess the efficacy of a new product, free of chemical insecticides but with a physical effect. This product contains components whose antilice efficacy has already been demonstrated, as well as Andiroba oil which asphyxiates the lice and Quassia vinegar which dissolves the chitin of the nits (they are then inactivated). 30 patients with head lice infestation, aged 3–39 years, applied the treatment one to three times, 5 days apart. Cure was defined as the absence of live lice after 5, 10, or 14 days, and symptoms are usually associated with infestation. Easiness and safety of the treatment were assessed by the patients and/or their parents. Overall cure rates were 20% on D5 after one treatment, 37% on D10 after two treatments, and 90% on D14 after three treatments. Symptoms such as itch, scalp dryness, redness, and flakiness rapidly diminished. This treatment seems to be a beneficial addition or a valuable alternative to existing treatments, considering the total absence of chemical insecticides, the absence of drug-resistance induction in head lice, the absence of major toxicological risks compared with usual pediculicides, and the favourable patient use instructions.

## 1. Introduction

Head lice (Pediculus humanus capitis) are wingless insects which are only found as parasites on humans and which nourish themselves solely from blood [1]. The head louse lays long pale gray to reddish-brown oval-shaped eggs (nits), size 0.3–0.8 mm. The female attaches the eggs to the hair, about 1–3 cm from the scalp, at the neck or behind the ears by preference, using strong water-proof cement. In one day they can lay up to 10 eggs, giving a life span potential of up to 300 eggs in their life time (30 to 40 days) [2, 3]. The larvae hatch within 7–10 days into a nymph or immature louse, which will die if a human blood meal is not acquired within 24 hours, and 8–10 days later are sexually mature. Thus a new generation is born within 18 to 24 days [4].

Agents currently approved by the US Food and Drug Administration for the treatment of head lice infestation are lindane, malathion, and permethrin crème rinse. Because of the development of resistance, other treatments have been considered such as trimethoprim, sulfamethoxazole, malathion, ivermectin and carbamyl, as well as combinations of these agents [5]. Today, the main treatments used are chemical agents such as pyrethrin (0.3%), malathion (organophosphate, M96), carabaryl, and permethrin (synthetic pyrethroid) [4, 6, 7]. The reference drug is usually permethrin [8, 9], which has been found to have the greatest efficacy and widest margin of safety, although the usual frequency of local irritative symptoms observed with permethrin ranges from 2.1% to 5.9% [10]. In the US, Lindane (1%, an organochloride) is now designated a “second-line” treatment, meaning it can only be prescribed when other “first-line” treatments have failed or cannot be used [11]. In Europe, lindane-containing medications have been removed from the market since 2007.

Moody and Ritter have underlined in 1989 [12] that the relatively high permeability of the forehead is important to note when considering that these insecticides are applied to the scalp for scabies control and that the human scalp and forehead have similar permeability. In order to decrease the toxic risk, some authors have already shown the interest to shorten the duration of exposure to the treatment to reduce its skin absorption [13].

Thus even if they are generally considered safe for occasional use, these treatments may pose a greater risk of direct or cumulative toxicity if used repeatedly and frequently [14–16]. Additional reasons to investigate alternative products are failure of current chemical treatments to kill embryos in eggs, environmental and food safety, and potential toxicity of the chemical pediculicides. There is a real need for compounds effectively killing adult lice and eggs by new modes of action. The benefit of essential oils in the control of head lice has been quoted since ancient times but few clinical studies have been published. Veal [17] tested *in vitro* the potential effectiveness of many essential oils and suggested an association of essential oil/vinegar/water to improve efficacy [17]. The combination of vinegar and water has been shown to loosen the hold and enhance removal [4]. A new approach, killing head lice by suffocation, has been described by Pearlman [18], who reported excellent clinical results with a so-called dry-on suffocation-based pediculicide (DSP; [18]). It was also shown in 2007 that dimethicone killed lice (*in vitro*) within 5 minutes, leading to a possible mode of action of low viscosity silicones by penetration into the spiracles and causing asphyxia and death [19]. Several promising reports have been subsequently published introducing new topical head lice compounds based on this mode of action [20–22].

The new product under study has been developed to treat hair and scalps afflicted by nits and head lice of children over 3 years of age and adults. It does not contain any chemical insecticide but rather has a physical effect. It is an entirely natural product (activates extracted from plants, no silicone) combining two active principles with effects on both nits and lice at the same time. The lice are asphyxiated by Andiroba oil. The Quassia vinegar dissolves the chitin of the nits, which are then inactivated. Additional components with demonstrated antilice efficacy are also included (Cocos nucifera, Thymus vulgaris; [23, 24]).

The objectives of the study were to assess the efficacy of this product as well as the time needed to cure the patients. Up to now, this product was used on D0 and applications repeated after 7 or 14 days if necessary. Buxton [25] has shown that a treatment is more effective if it is repeated after 10 days because eggs hatch 6 to 10 days after oviposition. This allows to eradicate the maximum number of lice on the head between the two treatments and while the lice are hatching from the eggs, an additional treatment on day 5 is recommended. This study was conducted in order to test if applications on D0, D5, and/or D10 would be sufficient to cure the patients within 14 days (last visit).

## 2. Material and Methods

This was a monocentre open interventional study of the efficacy and safety of a new product for treatment of head lice (Rausch Laus-Stop; Rausch AG Kreuzlingen, 8280 Kreuzlingen, Switzerland). 30 patients (29 female and one male patient aged 10.1 ± 7.2 years old (range 3 to 39 y; including 2 adults 25 and 39 y)) were recruited. The volunteers or one of the parents (or responsible relative) signed an informed consent after having carefully paid attention to the modalities and the aim of the study. A specific information note was prepared for the children in order to explain to them the aim and the main principles of these studies. It was asked to family members or fellow pupils who were in close contact to people with head lice to protect themselves from a possible lice infestation and to use a shampoo provided by the sponsor to wash their hair at home (RAUSCH Willow Bark Shampoo; Rausch AG Kreuzlingen, 8280 Kreuzlingen, Switzerland). The same shampoo was also provided to the patients. Clothing, bedding, brushes, combs, and personal items had to be deloused as well (by washing articles on a hot cycle or by deep-freezing).

Exclusion criteria were subjects affected by scalp disorders, history of irritation or sensitivity to pediculicides or hair care products, treatment with a pediculicide within 4 weeks prior to the study, having used hair dyes, bleaches, permanent wave, or relaxing solutions within the past 2 weeks or during the study. The study was approved by the local Ethics Committee (CPP Est II) and was notified to the French Authorities AFSSAPS. 

To assess the efficacy of the product accurately all patients were put in the same conditions: they received a shampoo to wash their hair at home, after every visit in the Dermatology Department and one or two times a week. (RAUSCH Willow Bark Shampoo; Rausch AG Kreuzlingen, 8280 Kreuzlingen, Switzerland). For patients with long hair, a detangling spray was also provided (“Rausch Herbal Detangling Spray”; Rausch AG Kreuzlingen, 8280 Kreuzlingen, Switzerland). It was applied on the 10-centimeter lower end of the hair (without rinsing) to make combing easier. These products were the only ones allowed as hair treatments during the study and were used according to the instructions of the manufacturer. 

The head lice treatment was performed every five days until cure by applying generously over the whole scalp, completely covering the hair roots, especially behind the ears and on the neck after ensuring that the scalp and hair were dry. The product was left on for 45 min, whereas if needed the head could be covered with a protective cap. This was the case for children, who could then freely move during this time. Thereafter, the product was rinsed off with plenty of warm water. The nits were carefully combed out and the hair was dried.

The efficacy of the treatment was judged by the disappearance of head lice and nits after one (D0), two (D0 and D5), or three applications (D0, D5 and D10). The last visit was scheduled on D14.

The primary outcome measure was the cure rate, defined as the percentage of patients cured after application of the treatment. Determination of the cure rate was performed by the investigator on the basis of visual inspection for viable nymphs, hatching nymphs, and adult lice (with a ×10 magnifying lens), using a head lice detection comb. The lice were counted and the infestation was scored as none (= 0), light (<10 lice) (= 1), moderate (between 10 and 20) (= 2), or severe (>20 lice) (= 3). A patient was cured if the score was 0 (no live lice) or failed if the score was 1 or higher (one or more live lice).

The secondary outcome measures were as follows.

- Intensity of pruritus on a Visual Analogical Scale (VAS).
- Clinical scoring of dryness, redness, irritation, and flakiness (0 = absence, 1 = light, 2 = moderate, 3 = severe).
- Clinical scoring of irritation, discomfort, and itching in order to assess the side effects of the product (0 = absence, 1 = light, 2 = moderate, 3 = severe).
- In case of children being treated, at each visit the parent's opinion was recorded regarding the workload required to perform the treatment. The parents were also questioned about any irritation, discomfort, embarrassment, or other symptoms associated with the use of this treatment.

## 3. Results

3 patients did not come back for the visit of D10. Two of them did not come because they felt cured (the D5 visit suggested that they were), and an exam finally performed on D14 showed that they were indeed cured. Two further patients did not come back on D14 because they were cured on D10 and one further patient did not come back without providing any explanation.

Analyses were performed on Per Protocol as well as on Intend to Treat population, that is, patients totally withdrawn were considered as not cured, and missing data on D10 were replaced as follows: if patients were scored cured on D5 and D14, they were considered cured on D10, if not, the worst score (from D5 or D14) was taken for D10.

**Figure 1:**
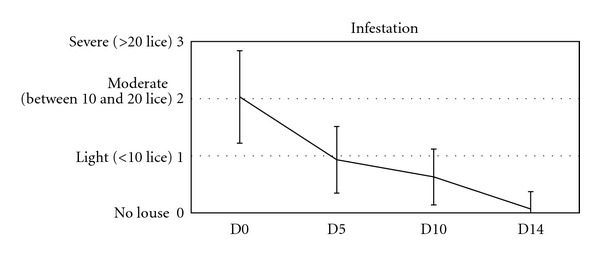
Patients' infestation over treatment period (mean ± standard deviation).

Figure 1 and Table 1 show the primary outcome (cure rate).  Table 2 describes the evolution of the cure rate for the whole population (ITT) or the Per Protocol (PP). After one product application (D5 score), 6 patients were cured (score 0). After two applications (D10 score), 11 patients were considered to be free of live lice (37%) and the other showed only light infestation. However, nits were still present in 93% of the evaluated patients, but the absence of re-infestation on D14 suggests that these nits were dead. Finally, after three applications (D14 score), a total of 27 patients (90%) were free of lice. 

One patient showed a new infestation on D14. This patient had no lice on D10 but was treated because some nits were still present. This reinfestation could be explained by transmission from her sister who also participated in the study and was still infested on D10.

Clinical scoring of symptoms usually associated with lice infestation such as itch, scalp dryness, redness (indicative of a possible inflammation), and flakiness rapidly diminished on treatment. Itch intensity decreased considerably from 7.3 to 3.7 in five days; and then disappeared in almost 60% of the patients (data not shown). On Day 14, only two patients were still experiencing a significant pruritus although one of these two patients presented no lice (but it could be explained by the fact he had dry hair). All other objective and subjective associated symptoms were similarly considerably decreased and almost disappeared on Day 14.

All patients found the infestation decreased or disappeared during the treatment. Three patients complained of slight scalp itching or exhibited head scratching after one or two applications. Three adverse events occurred, which were not attributable to the treatment.

## 4. Discussion

Up to now, products used in the control of head lice, including DDT and organochloride insecticides, may be considered relatively toxic and moreover, problems of toxicity and resistance have occurred so that the use of these products had to be discontinued [25]. In the last decades, as a result of extensive use, resistance to pediculicides with a neurotoxic mode of action has increased, particularly to permethrin [26–34]. Moreover, cross-resistance between some products such as permethrin and DDT or Bioallethrin has appeared. Permethrin has been used almost exclusively for louse control during some times [2]. Although pyrethrins were nearly 100% effective in the 1980s, recent studies suggest that this efficacy has decreased up to 50% [32]. Like pyrethrins, clinical failure rates of more than 50% have been reported with permethrin [26]. Similarly, Mumcuoglu et al. have reported that a 4-fold decrease in susceptibility to permethrin at the LT50 level was observed between 1989 and 1994 in Israel [35]. Resistances have been also reported to DDT, lindane, pyrethrins, permethrin, malathion, and carbaril. 

This phenomenon now extends worldwide and several possible resistance mechanisms have been reported [36, 37]. Bouvresse et al. have suggested that the uncontrolled use of pyrethroids should have induced the selection of lice having homozygous kdr mutations [38]. They have demonstrated in a study completed in 74 elementary schools in Paris in 2012 that 98.7% of the tested lice had such mutations. Even if the presence of these mutations may not correlate with treatment failure, an already strongly established insecticide has been revealed. Many factors may play a role: for example the residual activity of permethrin is still sufficient to kill lice 2 weeks after application. This implies that lice are exposed also during a significant period to sublethal doses of insecticides, exerting a strong selection pressure for insecticide resistance. Carbamates and organophosphorus preparations such as malathion have then be used with the advantages of being ovicidal as well as insecticidal, in time the head louse has developed resistance to these newer agents [39]. Now combinations of many insecticides are used [40]. Considering the composition of the test product, which is free of chemical insecticides and acts physically on lice (asphyxiated by the Andiroba oil) and nits (Quassia vinegar damages the chitin of the nits), the results of the study are very interesting. It should not lead to any resistance phenomenon and bears a far lower toxic risk than comparable insecticidal treatments.

In addition, treatments are often a burden for the patients and their family. Some treatments must remain a long time on the scalp before being washed out (e.g., 12 hours for malathion). Mumcuoglu [2] mentioned that suffocating agents such as olive, soya, sunflower and corn oils, hair gels, and mayonnaise are able to kill a significant number of lice only if they are applied in liberal quantities for more than 12 hours [2]. Asphyxiating treatments with silicone oil [21, 31, 41] require to be left to dry naturally for 8 hours then washed with a commercial shampoo. This has to be compared with a maximum of 3 times 45 min every 5 days with the test product, which therefore may be considered much more patient friendly and should ensure better compliance. This is important for curing infestation. 

On the whole, this cosmetic treatment has thus demonstrated its interest, with an efficacy which is similar or better than standard insecticidal treatments [27, 36]. This new scalp and hair treatment against head lice is free of chemical insecticides, contain natural ingredients, and act physically on lice. Its side effects are unusual: in exceptional cases there may be the occurrence of redness, dry skin, or flakiness. In comparison with the leader products, the costs of the treatments are customary in the market, with additional benefits for the scalp and without detrimental influence on the hair. It has shown a good efficacy after one, two, or three applications 5 days apart over a whole treatment period of 14 days. Three treatments were necessary for complete relief in severe cases. The symptoms associated with lice infestation such as itch, scalp dryness, and flakiness were rapidly and greatly decreased by the treatment. 

It may therefore be concluded that this new treatment is a beneficial addition or a valuable alternative to the existing ones, considering the total absence of chemical insecticides, the absence of drug resistance induction in head lice, the absence of major toxicological risks related to classic pediculicides, and the favourable patient use instructions. This should be particularly beneficial in children, who constitute the majority of the population to be treated for infestation with head lice.

## Figures and Tables

**Table 1:** Patients' cure rate (%) after treatment.

Infestation (*n* = 30)	D0	D5	D10	D14
0 (no louse)	0 (0%)	6 (20%)	11 (37%)	27 (90%)
1 (light/<10 lice)	9 (33%)	20 (67%)	19 (63%)	3 (10%)
2 (moderate/between 10 and 20 lice)	11 (37%)	4 (13%)	0 (0%)	0 (0%)
3 (severe/>20 lice)	10 (30%)	0 (0%)	0 (0%)	0 (0%)

**Table 2:** Cure rates, defined as absence of live lice for the intent to treat population (ITT) as well as per protocol (PP).

Infestation	ITT		PP	
	Cured/total	% (95% CI)	Cured/total	% (95% CI)
D5	6/30	20 (9.5–37.3%)	6/30	20 (9.5–37.3%)
D10	11/30	37 (21.9–54.5%)	9/27	33.3 (18.6–52.2%)
D14	27/30	90 (74.4–96.6%)	25/27	92.6 (76.6–98.0%)
